# Controlled glucose consumption in yeast using a transistor-like device

**DOI:** 10.1038/srep05429

**Published:** 2014-06-25

**Authors:** Yang Song, Jiapeng Wang, Siu-Tung Yau

**Affiliations:** 1 Department of Electrical and Computer Engineering, Cleveland State University, Cleveland, Ohio 44115, USA; 2 The Applied Bioengineering Program, Cleveland State University, Cleveland, Ohio 44115, USA

## Abstract

From the point of view of systems biology, insight into controlling the functioning of biological systems is conducive to the understanding of their complexness. The development of novel devices, instrumentation and approaches facilitates this endeavor. Here, we show a transistor-like device that can be used to control the kinetics of the consumption of glucose at a yeast-immobilised electrode. The gating voltage of the device applied at an insulated gating electrode was used to control both the rate of glucose consumption and the rate of the production of ATP and ethanol, the end-products of normal glucose metabolism. Further, a correlation between the glucose consumption and the production of ethanol controlled by the gating voltage was observed using two different forms of the device. The results suggest the relevance of glucose metabolism in our work and demonstrate the electrostatic nature of the device. An attempt to explain the effect of the gating voltage on the kinetics is made in terms of transfer of electrons from NADH to enzymes in the electron transport chain. This novel technique is applicable to general cells and the reported results show a possible role for electrostatic means in controlling processes in cells.

Controlling the processes or functioning of biological systems has profound implications in medicine and other applications^1^. An important biological process is the metabolism of glucose. Glucose metabolism refers to the cellular processes that convert glucose to energy for cell utilization^2^,^3^. Although metabolism has been well studied, there is a recent renewed interest in this subject focusing on its central role in areas of cell biology, physiology, medicine and synthetic biology^4^. Two examples from these areas show the importance of the regulation of glucose metabolism. Compared with normal cells, cancer cells consume much more glucose and mainly process it through aerobic glycolysis, as described by the Warburg effect^5^. The theory of quantum metabolism^6^ elucidates the differences in metabolic rates between normal cells and cancer cells using electron transit time, which describes the turnover time of redox reactions. The intricate link between metabolic regulation and cancer formation and progression is currently a focus in cancer research. Metabolic engineering is another metabolism-related area of current interests, where attempts are made to use cellular metabolic pathways in yeast or bacteria to synthesise compounds or fuels that are difficult or expensive to produce by other means^7^,^8^.

An important component of metabolism is cellular electron transport. The feasibility of controlling electron transfer in biological systems using a gating voltage was demonstrated in the reduction of hydrogen peroxide (H_2_O_2_) at an electrode immobilised with microperoxidase-11^9^, showing controlled kinetics of the bio-catalytic system. Engineered electron transport was achieved in *E. coli* to produce hydrogen using elimination of competing reactions, engineering of protein interaction surfaces, and protein fusion or scaffolding^10^. The present work shows that the rate of glucose consumption in the presence of yeast (*Saccharomyces cerevisiae*) that is in contact with an electrode can be electrostatically controlled using a transistor-like device. Experimental observations show that the gating voltage of the device can be used to accelerate or decelerate the depletion of glucose in samples, depending on the polarity and magnitude of the voltage. Additionally, the production of adenosine triphosphate (ATP) and ethanol, which are the end-products of glucose metabolism, can also be controlled using the gating voltage. The results of this work suggest that, to a certain degree, the kinetics of the glucose metabolism in the yeast can be controlled using the gating voltage of the device. As shown in Figure 1, the experimental setup consists of a conventional three-electrode electrochemical cell modified with additional gating electrodes, which are internally connected and electrically insulated from the electrochemical cell system, for applying the gating voltage V_G_ to the working electrode. Yeast is immobilised on the working electrode and the cell contains a glucose solution. Note that V_G_ causes no current in the circuit containing the gating electrode. An explanation of this electrostatic technique in terms of previous results is given in Supporting Information.

## Results

### Cyclic voltammetric measurement of glucose oxidation

**Figure 1:**
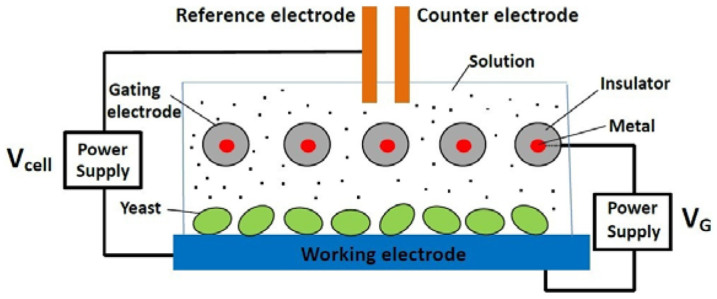
Experimental system. A conventional three-electrode electrochemical cell is modified with additional gating electrodes for applying a gating voltage V_G_ to the working electrode, upon which yeast cells are immobilizsed. V_cell_ is the cell potential. The gating electrodes are coated with an insulator with their metallic parts electrically connected.

**Figure 2:**
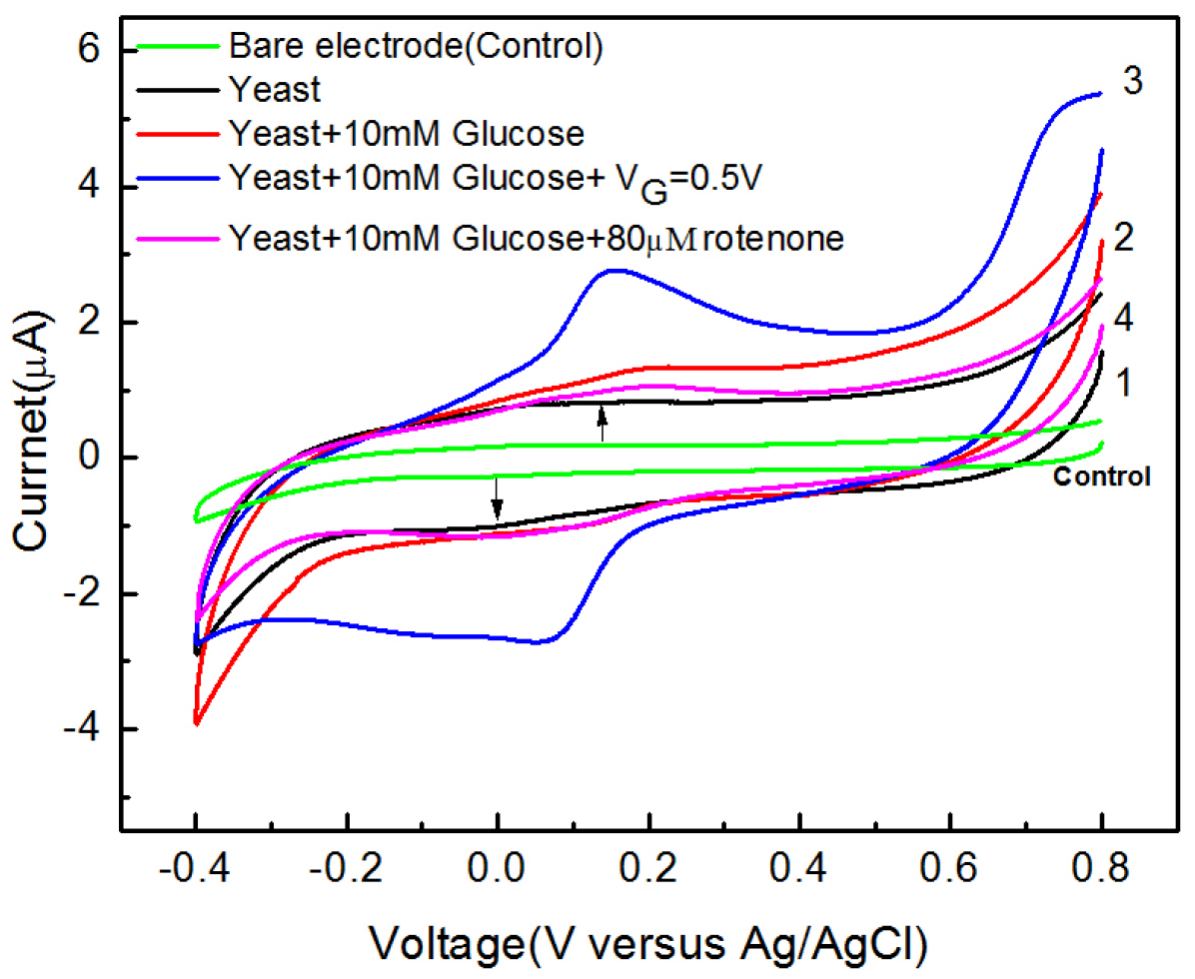
CVs showing the yeast-induced oxidation of glucose. The arrows indicate the redox peaks of yeast.

We first show that oxidation of glucose takes place at the yeast-immobilized electrode and the oxidation can be controlled by V_G_. Cyclic voltammetry of glucose was performed using the system shown in Figure 1 to show the yeast-induced oxidation of glucose. The cyclic voltammograms (CVs) in Figure 2, except the control CV, were obtained using a yeast-immobilised graphite electrode under different conditions. CV1 was obtained in phosphate buffered saline (PBS) whereas CV2 was obtained with glucose added to the PBS. CV 1 shows a pair of weak redox peaks indicated by the arrows with a formal potential at 50 mV vs. Ag/AgCl. Previous voltammetry of yeast showed similar peaks^11^. Comparing CV2 with CV1 shows increased anodic current, indicating the oxidation of glucose by the yeast. CV3 shows further increase in the oxidation current and enhanced redox peaks caused by the application of a positive V_G_. The control CV was obtained using a bare electrode with and without glucose in the solution. The control CV is flat and does not change with V_G_ and different glucose concentrations. The CV results suggest that the observed glucose oxidation was due to the presence of yeast. CV4 was obtained with 80 μM of rotenone, an electron transport chain inhibitor, added to the glucose solution with and without V_G_.

### V_G_-dependent glucose consumption

**Figure 3:**
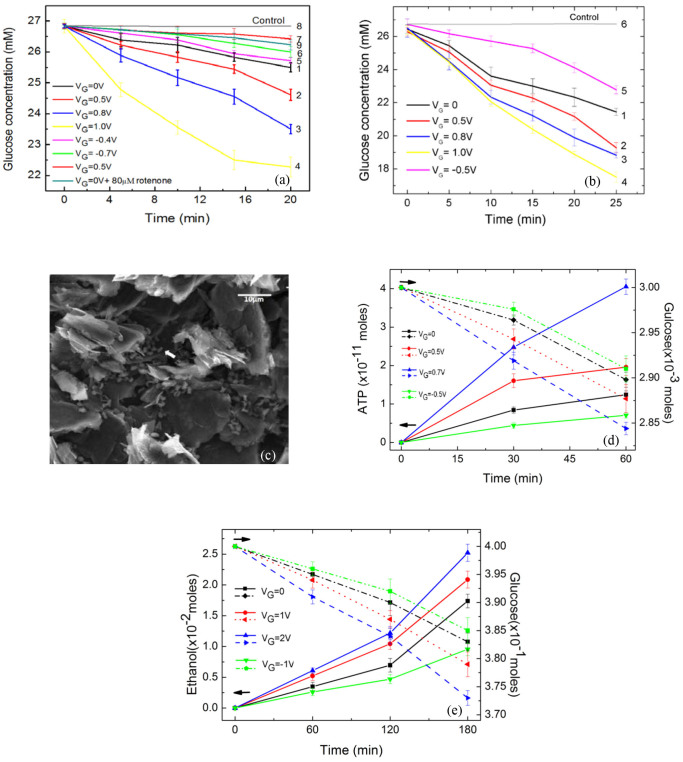
Voltage-controlled glucose metabolism in yeast. (a) and (b) show the V_G_-dependent change in glucose concentration under the aerobic condition and the anaerobic condition, respectively. (c) SEM image of yeast-immobilised electrode before electrochemical processing, showing yeast cells as indicated by the arrow. (d) and (e), respectively, show the simultaneous measurements of V_G_-controlled glucose consumption and production of ATP and ethanol. Error bars: S.D.

A more direct way of showing the oxidation of glucose is to use the system in Figure 1 to monitor the change in the glucose concentration of samples for different values of V_G_. Samples of glucose in PBS (26.8 mM, 2 mL) were processed using the system in Figure 1 at room temperature (23°C) with yeast-immobilised graphite electrodes polarised at V_cell_ = 0.6 V vs. Ag/AgCl for a total time of 20 min. Aliquots of 5 μL were taken from the processed samples every 5 min to be measured using commercial glucose test strips and a glucose metre, whose measuring range is 20–600 mg/dL (1.11–33.33 mM). The curves in Figure 3 (a) show the V_G_-dependent changes in glucose concentration under the aerobic condition. The curves were obtained with yeast-immobilised electrodes prepared under identical conditions. Curve1, obtained with V_G_ = 0 V, shows a gradual decrease in glucose concentration spanning the 20 min period. The effect of a positive V_G_ appears to be causing faster decreases in glucose concentration. As V_G_ is increased, the decrease in glucose concentration occurs at progressively faster rates as indicated by Curves 2–4. When the polarity of V_G_ is reversed, the rate becomes progressively slower than that of Curve1 as indicated by Curves 5–7. Curve 8 is the control trace obtained using a bare electrode, showing no change in glucose concentration. Curve 9 was obtained with 80 mM of rotenone and V_G_ = 0V. Figure 3 (b) shows the results obtained under the anaerobic condition (achieved by purging solution with dry N_2_ for 1 hour). The curves in Figure 3 (b), obtained under otherwise identical conditions, show similar trends as in the aerobic case.

The measurements of this work were carried out in the lag phase of yeast budding (2–10 hours)^12^ to avoid yeast reproduction. The scanning electron microscopy (SEM) image in Figure 3(c) shows a selected area of a graphite electrode before carrying out the electrochemical processing of a glucose sample. The 3 μm × 1 μm grain-like structures are yeast cells. SEM images of the same area after 120 min of electrochemical processing in glucose (image not shown) appear to be identical to the one in Figure 3 (c), suggesting that the faster consumption of glucose was not caused by yeast reproduction. Using SEM images, the number of yeast present in the measurements in Figures 3 (a) and 3(b) is estimated to be 6 × 10^8^.

### V_G_-dependent production of metabolic end-products

To provide basis for the elucidation of the observed V_G_-dependent glucose consumption, adenosine triphosphate (ATP) and ethanol, the end-products of the normal metabolic processes, were probed for possible changes in their amounts caused by V_G_. Aerobic metabolism of glucose is the dismantlement of glucose by the three component processes of metabolism, namely, glycolysis, Krebs cycle and the electron carriers' traversing the electron-transport chain, in the presence of oxygen. ATP is synthesised in the three processes. In cells, ATP is produced following the overall simplified reaction under the aerobic condition: Glucose + oxygen + specific sequence of reactions controlled enzymes & coenzymes ——> carbon dioxide + water + ATP. This reaction depends on the activities of three coenzymes: nicotinamide adenine dinucleotide (NAD); nicotinamide adenine dinucleotide phosphate (NADP); and flavin adenine dinucleotide (FAD)^3^.

Luminescence assay of ATP in yeast cells immobilised on a carbon cloth electrode and electrochemically processed in a 100 mM glucose solution (30 mL) was performed to reveal possible changes in the amount of ATP as a function of V_G_. Figure 3 (d) shows the simultaneous measurements of the glucose consumption and the production of ATP in the same sample under the aerobic condition. A correlation between the decrease in the amount of glucose and the increase in the amount of ATP is present during the 60-minute processing period. The correlation persists for zero, positive and negative V_G_.

Ebulliometry of electrochemically processed glucose samples was performed to monitor the possible production of ethanol under the anaerobic condition. The curves in Figure 3(e) show the simultaneous measurements of glucose consumption and production of ethanol occurring in 400 mM glucose samples (1 L) with and without V_G_ at different times during a 180 min (3-hour) period at room temperature (23°C). The measurements are expressed in units of moles, calculated using the simultaneously measured concentrations in the same sample. The curves show that positive V_G_ leads to faster glucose consumption and faster production of ethanol, whereas negative V_G_ resulted in glucose consumption and ethanol production below their respective levels obtained with V_G_ = 0. Figure 3 (e) shows that a correlation exists between the decreasing glucose amount and the increasing ethanol amount for zero, positive and negative V_G_. The production of ethanol in the presence of glucose and yeast, a direct result of fermentation, can be used as an indirect way of showing the fact that at least the fermentation part of the yeast's activity was present at the end of 3 hours' operation of the electrostatic-electrochemical system.

## Discussion

The results presented above clearly indicate that V_G_ controls the glucose consumption and the production of ATP and ethanol. An analysis of the results provides clues to possible causes of the observed effects and additional insights. Figure 2 shows that glucose oxidation occurred at the yeast-immobilised electrode. Figures 3 (a) and 3 (b) show that the glucose consumption in the presence of a yeast-immobilised electrode was controlled by V_G_. The results of control experiments show that the oxidation and consumption of glucose was due to the yeast since bare electrode did not cause these effects. Figure 3 (d) shows the simultaneous measurements of glucose consumption and production of ATP in the same sample under the aerobic condition. The amount of glucose in the sample decreases while that of ATP increases, indicating a V_G_-dependent correlation between the two processes. The disparity between the extreme glucose-to-ATP ratio shown in Figure 3 (d) and that of the standard ratio (1:36) could be the result of the fact that the measurements was made in the lag-phase.(See below) Nevertheless, the observed correlation indicates that the two processes are governed by a specific process.

Figure 3 (e) shows the simultaneous measurements of glucose consumption and production of ethanol in the same sample. In the absence of V_G_, glucose consumption and ethanol production occur as a function of time, i.e. the amount of glucose decreases while that of ethanol increases. The most obvious explanation for this observed correlation is the fermentation of glucose induced by yeast. Note that fermentation is a process of metabolism. Further, as shown in Figure 3 (e), the glucose consumption occurs at faster rates as a positive V_G_ is applied, an effect also observed in Figure 3 (b). At the same time, the production of ethanol also occurs at a faster rate as a positive V_G_ is applied. Figure 3 (e) shows that the correlation between the glucose consumption and ethanol production is controlled by V_G_. Therefore, the results of Figure 3 suggest the possibility of V_G_-controlled anaerobic glucose metabolism.

The correlation shown in Figure 3 (e) is expressed in the number of moles of the respective substances. The pair of curves obtained for V_G_ = 0 shows that, at the end of the 180 min period, glucose undergoes roughly a decrease of 0.17 × 10^−1^ mole, while ethanol shows a gain of 0.175 × 10^−1^ mole. Considering the 1:2 glucose-to-ethanol ratio of the normal fermentation process, the observed 1:1 glucose-to-ethanol conversion indicates a hampered fermentation process. In fact, all of the pairs of curves in Figure 3 (e) show an approximate 1:1 glucose-to-ethanol conversion. A possible explanation is that the measurements were made in the lag-phase of yeast growth, where the yeast underwent preparation for reproduction and its normal functioning was not fully restored. However, the fact that both glucose consumption and ethanol production increase with V_G_ indicates that V_G_ was able to affect the functional process that was producing ethanol. The disparity between the glucose-to-ATP ratio shown in Figure 3 (d) and that of the standard ratio may also be attributed to the lag-phase.

There are additional observations that add to the credibility of glucose metabolism. Rotenone, an electron transport chain inhibitor that blocks the chain between NADH dehydrogenase (Complex I) and ubiquinone, was used in the glucose oxidation and consumption experiments to reveal its inhibiting effect. CV4 in Figure 2 shows that rotenone significantly reduces the oxidation current of glucose. In Figure 3 (a), Curve 9 obtained in the presence of rotenone shows reduced glucose consumption compared to Curve 1. The inhibiting effect suggests the relevance of metabolism in the present work. Furthermore, comparing the glucose consumption curves in Figures 3 (a) and 3 (b) shows that under identical conditions the anaerobic process shows faster consumption rates than those for the aerobic case. This effect is qualitatively consistent with the fact that the rate of glucose consumption is higher under anaerobic conditions than that for the aerobic case due to the fact that the anaerobic process is associated with a low energy yield^13^.

Although the results presented above suggest the presence of metabolism, which can be controlled using V_G_, it should be noted that other effects might also be involved in the experiment. In general, electric current can cause chemical or mechanical changes in the sample solution. For example, glucose may be forced into the yeast so that the glucose concentration becomes lower or biochemical reactions can be caused by effects such as transporters and ion channels. Therefore, the results obtained may be due to factors in addition to metabolism.

**Figure 4:**
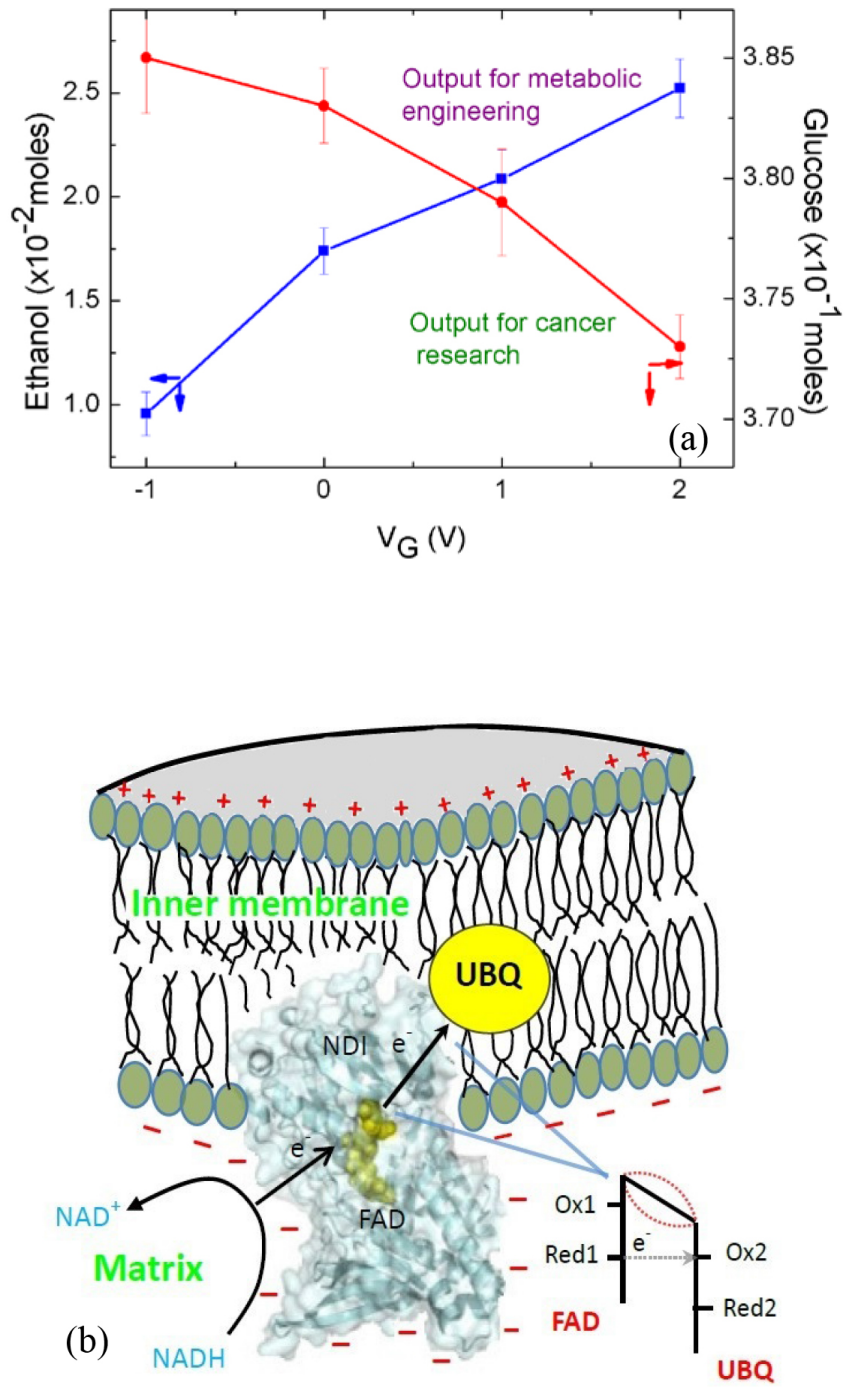
Characteristics of the metabolic transistor. (a) Transfer characteristics of the transistor-like device. The output quantities of the transistor are dependent on V_G_. (b) A plausible scenario for the transfer of electrons from NADH to NDI and the subsequent tunnelling through the enzyme to UBQ. The electric field caused by the V_G_-induced charge separation modulates tunnel-barrier height. “ox” and “red” respectively are the oxidized and the reduced energy levels of the electro-active regions.

A reexamination of Figure 1 and Figure 3 indicates a resemblance between the field-effect transistor (FET) and the experimental system shown in Figure 1 with its insulated gating electrode being used as an electrostatic means to control the output quantities. In the present work, the process that converts glucose to ATP and ethanol is analogous to the channel of the FET; V_G_ can be viewed as the input quantity and the glucose consumption, the production of ATP and ethanol are the output quantities. Figure 4 (a) shows two examples of the transfer characteristics of the transistor-like device. The output quantities are taken from the values at 180 min in Figure 3 (e).

How does V_G_ alter the conversion process? Presently a clear-cut description of the mechanism of the observed effects is not available. However, if metabolism is employed to account for the conversion process, a plausible scenario can be put forward for further investigation. Since the gating electrodes are electrically insulated, the modification produced by V_G_ to the metabolic processes must be of an electrostatic nature. The different pathways in yeast all involve redox reactions catalysed by redox enzymes. For example, the redox reaction of the NAD^+^/NADH redox couple is catalysed by different dehydrogenases in glycolysis, the Krebs cycle and the electron transport chain^3^.

Previously, it was demonstrated that a gating voltage can be used to control the electron transfer between a redox enzyme and an electrode^9^,^14^. The effect, demonstrated with the glucose oxidase-glucose system and the microperoxidase-H_2_O_2_ system, was attributed to the redistribution of charges at the solution-electrode interface induced by the gating voltage so that an electric field is set up to modulate the electron tunnel barrier, which is the protein network between the active site of the enzyme and the electrode. This mechanism was further investigated by studying the effects of parameters and reaction kinetics in order to provide evidence for the proposed mechanism^15^. An explanation of this electrostatic technique in terms of previous results is given in Supporting Information. Based on these prior works, it is speculated that similar scenarios are also relevant to the present work. In particular, it is deemed possible that the rate of electron transfer associated with various redox enzymes in the metabolic processes can be modulated by electric fields induced in the interior of the cell. To this end, the system comprising NADH, the internal NADH dehydrogenase (NDI) and the ubiquinone (UBQ) in the electron transport chain is chosen only as an example for illustration purposes. Figure 4 (b) is a schematic diagram showing the passing of electrons from NADH to the yeast's NDI^16^ situated in the electron transport chain and the subsequent traversing of the electrons through the FAD center to the UBQ. Figure 4 (b) shows the V_G_-induced charges around the enzyme. The induced charge sets up an electric field, whose component opposite to the electron's movement through the tunnel barrier of the enzyme modulates the effective height of the barrier so that the electron transfer rate can be increased or decreased^17^,^18^. Similar modulated electron transfer may also occur with other systems of redox enzymes involved in the metabolic process, leading to faster production of end products. Figure 3 shows that, whereas positive V_G_ values enhance the featured effects, reversing the polarity of V_G_ diminishes the effects. This observation implies that the reversed V_G_ increased the tunnel barrier height and therefore provides evidence for the proposed mechanism.

**Figure 5:**
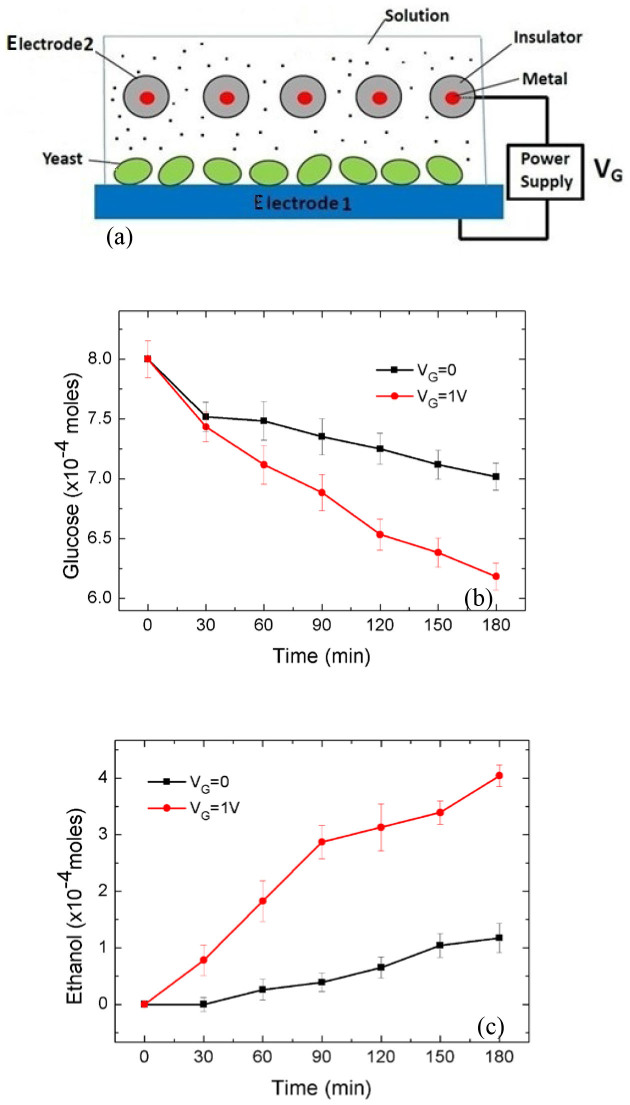
A two-electrode device and its glucose-ethanol conversion characteristics. (a) A schematic of the device. (b) The glucose consumption characteristics. (c) The ethanol production characteristics. The measurements in (b) and (c) were obtained with a 50 mm × 30 mm carbon cloth working electrode in a 30 mL glucose solution (100 mM). No current was observed in the two-electrode device during the entire process. These results confirm the electrostatic origin of the altered conversion processes.

To provide further support for the electrostatic nature of the observed effect of V_G_, the process of glucose-ethanol conversion was performed using a two-electrode system, as shown in Figure 5 (a), which consists of a yeast-immobilized carbon cloth electrode connected via a power supply V_G_ to an insulator-coated metallic wire. The two electrodes were immersed in a glucose solution and a dc voltage V_G_ was applied between them. Simultaneous measurements of glucose and ethanol concentrations were made with the same solution. No current was observed in the measurements. Figures 5 (b) and 5 (c) show that glucose consumption and ethanol production are accelerated due to the application of V_G_, both effects being similar to those shown in Figure 3 (e). Figures 5 (b) and 5 (c) unequivocally shows the electrostatic cause of the altered conversion processes.

The electrical communication between the yeast and the electrode is discussed as a final note. The oxidation currents shown in Figure 2 indicate that the yeast cells, while processing glucose, are able to pass electrons to the electrode. Since the thickness of the cell wall is about 100–200 nm^19^, the observed extracellular electron transfer requires special mechanisms. Among the several mechanisms that have been proposed for extracellular electron transfer^20^, the direct transfer of electrons via the interaction between intracellular electron carriers such as NADH and the trans-plasma membrane electron transfer (tPMET) system, a set of redox enzymes and proteins located in the plasma membrane^21^,^22^, is most relevant to the present work. The tPMET system consists of cytochromes and various redox enzymes such as NADH oxidase, providing redox activity of the membrane at specific sites.

The present work demonstrates the electrostatic control of glucose consumption and the production of the end-products of the normal glucose metabolism, namely, ATP and ethanol, in the presence of yeast immobilised on an electrode. Observation show that a correlation exists between the consumption and production and this correlation is also controlled electrostatically via the gating voltage V_G_ of an electrostatic-electrochemical system. Since V_G_ does not cause current in its circuit, a resemblance of the electrostatic-electrochemical system to the transistor is noticed. The results of this work suggest that V_G_ of this transistor-like device can be used as a convenient parameter to control the kinetics of the consumption of glucose and the production of ATP and ethanol in the presence of yeast immobilized on an electrode. Additional experimental observations, including the effect of an inhibitor, the difference between the aerobic and anaerobic glucose consumption and, most importantly, the simultaneous measurements of glucose and ATP and ethanol in the same sample using the three-electrode and the two-electrode devices, provide supporting evidence that the observed glucose consumption is due to metabolism in yeast and the metabolism can be controlled using V_G_ with the possible occurrence of other processes caused by the electrostatic-electrochemical operation. The interaction between NADH and enzymes in the electron transport chain is chosen as an example to illustrate a possible mechanism of the V_G_-controlled processes in terms of cellular electron transfer. This technique may find applications in cancer research and diagnosis and in metabolic engineering, where the kinetics of the production of substances can be controlled using a voltage. In fact, the V_G_-controlled ethanol production demonstrated here shows a possible role for external voltage in metabolic engineering.

## Methods

### Yeast preparation

Dried baker's yeast (*Saccharomyces cerevisiae*) purchased from Sigma Aldrich (YSC1) was cultivated for two hours at 30°C in a solution of deionized water, glucose and peptone, in which the concentration of dry yeast was 1 g/L.

### Electrode and electrochemical measurement system

Yeast-immobilised working electrodes were prepared by depositing a 0.1 ml drop of the yeast solution (after cultivation) on a 2 mm × 2 mm area defined using a mask on a pyrolytic graphite (PG) electrode and incubating the electrodes at room temperature for 2 hours. A conventional three-electrode electrochemical cell with a volume of 2 mL was used to perform electrochemical measurements. The yeast–immobilized electrodes were used as working electrodes. For the measurement of ATP and ethanol, carbon cloth was used as the electrode. A piece of carbon cloth was immersed in a yeast preparation solution as described above for two hours to allow the yeast, while being cultivated, to become adsorbed on the electrode. A commercial Ag/AgCl (3 M KCl) electrode was used as the reference electrode and a platinum wire was used as the counter electrode. A piece of 0.5 mm-diameter copper wire coated with a thin layer of insulator (enamel) was used as the gating electrode. The wire was bent to form multiple turns and attached on the graphite electrode next to the immobilized yeast. For the carbon cloth electrode, the wire was used to wind around the electrode. The electrochemical cell was driven by a commercial electrochemical controller (CH Instruments 660C). The phosphate buffered saline (PBS) used in this work was prepared using de-ionized water (18.2 MΩ-cm). All measurements were made with PBS at room temperature.

### Glucose measurement

BREEZE®2 blood glucose test strips and a BREEZE®2 blood glucose metre (Bayer Health Care, Mishawaka, WI) with a measuring range of 20–600 mg/dL (1.11–33.33 mM) were used to measure the concentration of glucose in samples. The metre was calibrated before use.

### ATP measurement

The assay of ATP was performed using luminescence method with the BacTiter-Glo™ Microbial Cell Viability Assay kit (Promega, Madison, WI). The luminescence was detected using a Victor3 Multilabel Plate Counter (Perkin Elmer) and displayed as relative light units (RLU). The first step of the assay was the lysis of the yeast cells (destruction of cell) using a chemical solution in order to release ATP from the interior of the cells. A fixed portion (2 mm × 2 mm) of the yeast-immobilised carbon cloth electrode that had undergone the electrochemical procedures was immersed in the lysis solution in a 200 μL microwell so that ATP became dissolved in the solution. Then, a second solution containing luciferin, luciferase and magnesium ions was added to the microwell. The oxidation of luciferin catalyzed by luciferase turned ATP to AMP with luminescence generated at 600 nm. The luminescent signal is proportional to the amount of ATP present. A standard curve of the counter was produced to convert the unit of ATP from RLU to molar concentration. The final assay solution had a pH of 7.2. The half-time of ATP in lysate is short and measured in seconds^23^ due to the presence of ATPase, which decomposes ATP into ADP. The assay solution deactivates ATPase to stabilize ATP so that measurements can be performed within a window of 30 min after the lysis procedure is completed. Each ATP measurement was made using a sample for a one-time use only.

### Ethanol measurement

The ethanol concentration of samples (%v/v) was measured using an ebulliometer (Dujardin-Salleron, Paris, France) at room temperature. The production of ethanol took place in a beaker, containing 1 liter mL of a glucose solution(400 mM). A 40 mm × 500 mm carbon cloth immobilized with yeast was used as the working electrode.

### Scanning electron microscopy

The field emission scanning electron microscope used to image the immobilized yeast on electrodes was made by Hitachi (FE-SEM 5000).

## Author Contributions

Y.S. and J.W. performed the experimental part of the work S.-T.Y. conceived the work, designed and directed the experiments, analysed the data and wrote the paper. All authors discussed the results and commented on the manuscript.

## Supplementary Material

Supplementary Information Controlled glucose consumption in yeast using a transistor-like device
