# Supplementary material for: Controlled glucose consumption in yeast using a transistor-like device
Source: Sci Rep. 2014 Jun 25;4:5429. doi: 10.1038/srep05429 (PMC4069707; doi:10.1038/srep05429)
Supplement: Supplementary Information — Controlled glucose consumption in yeast using a transistor-like device [file srep05429-s1.doc]

**Supporting Information**

**Controlled glucose consumption in yeast using a transistor-like device**

Yang Song, Jiapeng Wang & Siu-Tung Yau

Previously, it was demonstrated that a gating voltage can be used to control the electron transfer between a redox enzyme and an electrode^[S1, S2]^. The effect, demonstrated with the glucose oxidase-glucose system and the microperoxidase-H_2_O_2_ system, was attributed to the redistribution of charges at the solution-electrode interface induced by the gating voltage so that an electric field is set up to modulate the electron tunnel barrier, which is the protein network between the active site of the enzyme and the electrode. This mechanism was further investigated by studying the effects of parameters and reaction kinetics in order to provide evidence for the proposed mechanism ^[S3]^. In the present work, this electrostatic process is mentioned as a possible cause of the observed effects. In the following, a detailed account is given to explain the principle of this technique in relation to biosensors.

Redox enzymes are immobilized on the sensing electrodes of biosensors as sensing elements. The active sites of redox enzymes are embedded in a three dimensional polypeptide network. The isolation of the active site by the polypeptide network causes low level of interfacial electron transfer between the enzyme and the electrode of a biosensor, creating a fundamental limit on the sensitivity of amperometric biosensors. In general, the detection limit of conventional biosensors is in the micro molar (10^-6^ M) analyte range.

Figure S1 (a) is a schematic description of the detection system. It consists of a conventional three-electrode electrochemical cell with a cell potential V_cell_ connected between the working electrode and the reference electrode. Under the normal operation condition, an electric double layer is present near the surface of the working (sensing) electrode. The cell is modified with additional gating electrodes for applying a gating voltage *V_G_* between the gating electrode and the working electrode, upon which a redox enzyme is immobilized. *V_G_* modified the interfacial charge distribution. When *V_G_* is positive, additional negative charges are induced within the working electrode and additional positive ions in solution are induced at the solution-enzyme-electrode interface. Figure S1 (b) depicts the net interfacial charge distribution. Some positive ions are able to set up electric fields with the presence of the transferring electrons residing at the enzyme’s active sites (the red circles) as indicated in Figure S1 (b). The fields lower the potential energy experienced by the transferring electrons in the polypeptide region between the active sites and the electrode.

Figure S1 (a) Cross-sectional view of the field–effect bio-detection setup. Each elliptical structure represents an enzyme molecule. The enzyme’s active center is indicated by the smaller circle within a molecule. The gating electrodes are represented by the circular structures, which consist of a copper wire (the blue circles) and a thin layer of insulator (the shaded shells). *V_G_* is a voltage source used to produce an electric field at the interface between the sample solution and the working electrode. (b) Conceptual description of enzyme-electrode interface. The red dot is the active site of the enzyme. The double layer and induced charges are indicated. The induced charge set up the field within the enzyme. The enzyme catalyzes the oxidation of the analyte, resulting in electrons e^-^ transferred from the active site to the electrode. (c) The effect of the V_cell_ and that of the induced electric field is depicted using the interfacial electron energy profile.


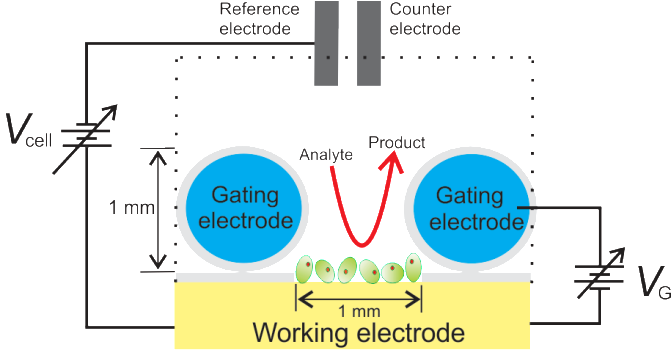


(a)


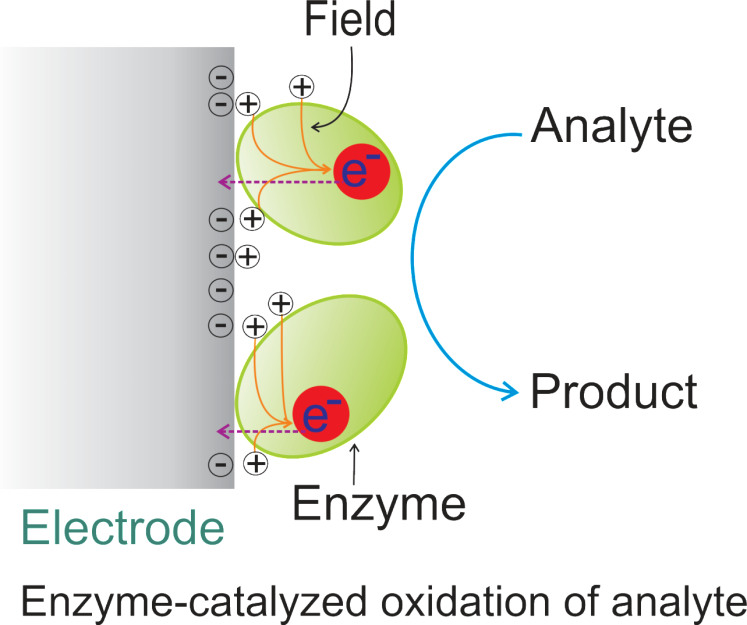

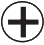

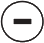


(b)


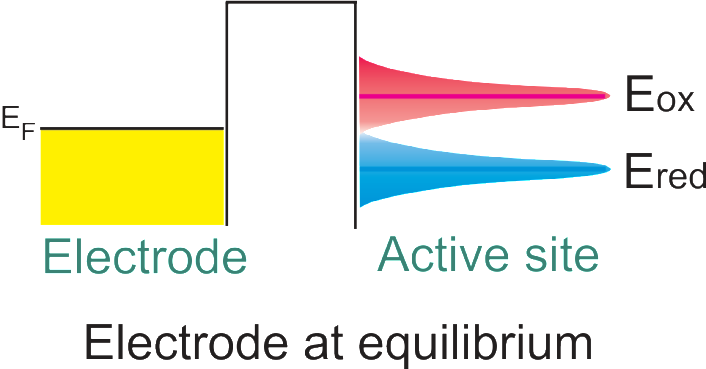

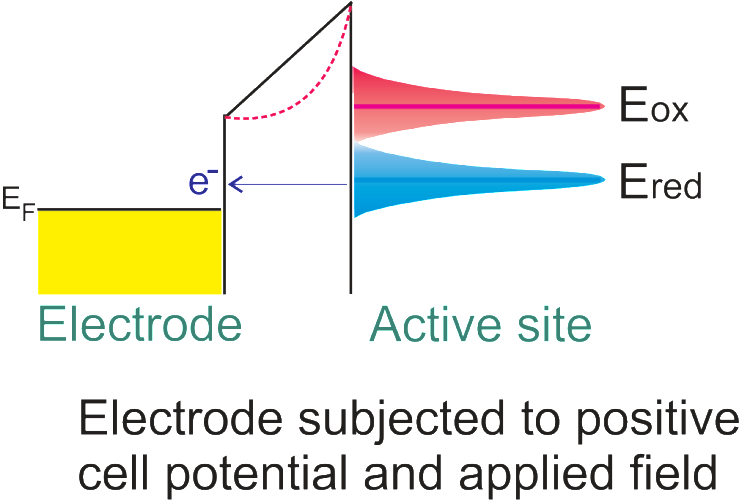


(c)

The signal current of a biosensor using immobilized redox enzyme as the sensing element is the result of quantum mechanical tunneling of electrons from the active site of the enzyme through the polypeptide tunnel barrier to the electrode. The energy profile of tunnel barrier can be modified by an electric field so that the tunneling rate is enhanced. As explained above (see Figure S1 (b)), the induced fields lowers the height of the tunnel barrier (the potential energy experienced by the transferring electrons) and therefore increases the electron tunnel rate and hence the current. The result of this process is an amplified signal current.

Figure S1 (c) shows a conceptual energy-band profile of the enzyme-electrode interface. At equilibrium, no electron transfer occurs between the active site and the electrode, since the most probable energy of the occupied quantum state of the active site, *E_red_*, is below the Fermi energy *E_F_* of the electrode. When the cell potential V_cell_ is raised, oxidation of the enzyme occurs as electrons are energetically allowed to be transferred from the *E_red_* to the electrode. The electrode-active site system can be considered as a acceptor-donor pair, and the electron transfer rate constant *k_et_* depends critically on the distance *d* between the electrode and the active site as *k_et_  exp(-βd)*. The exponential dependence of *k_et_* on *d* effectively diminishes electron transfer. However, the rate constant also depends on the value of the attenuation coefficient*, β,* which is proportional to the square root of the tunnel barrier height (*β*  (Ф_B_)^1/2^). When *V_G_* is turned on, the induced electric field distorts the top of the tunnel barrier (see the red dashed curve), reducing the effective height of the barrier and, therefore, resulting in a smaller value of *β* and therefore a larger value of *k_et_*. Thus, electron conduction in the nanoscale region between the active site and the electrode is enhanced, resulting in increased analyte oxidation current (amplified signal) and therefore lowered detection limit.

In the present work, the enzyme in Figure S1 (a) is replaced by the redox enzymes involved in the three component processes of metabolism, namely, glycolysis, Krebs cycle and the electron-transport chain.

Reference

[S1] Y. Choi, S.-T. Yau, *AIP Advances* **2011**, *1*, 042175.

[S2] Y. Choi, S.-T. Yau, *Anal. Chem.* **2009**, *81*, 7123-7126.

[S3] S.-T. Yau, Y. Xu, Y. Song, Y. Feng, J. Wang, *Phys. Chem. Chem. Phys.* **2013**, *15*, 20134--20139.
